# Optimization of amylase production by the biological control agent *Bacillus halotolerans* RFP74 using response surface methodology

**DOI:** 10.1186/s43141-023-00519-4

**Published:** 2023-05-19

**Authors:** Pelias Rafanomezantsoa, Samia Gharbi, Noureddine Karkachi, Mebrouk Kihal

**Affiliations:** 1 Department of Biological Science, Applied Microbiology Laboratory, University Oran 1 Ahmed Ben Bella, Oran, Algeria; 2 Department of Biotechnology, University of Science and Technology of Oran Mohamed Boudiaf, Oran, Algeria

**Keywords:** Antifungal, Amylase, Bacillus, Biocontrol, Optimization

## Abstract

**Background:**

Over the years, excessive use of chemical pesticides to control plant pathogens has caused environmental problems. Therefore, biological solutions such as the use of microorganisms with antimicrobial capacity become indispensable. To inhibit the growth of plant pathogens, biological control agents use different mechanisms, including the production of hydrolytic enzymes. In this study, the production of amylase, an enzyme important for the prevention and control of plant diseases, by a biological control agent *Bacillus* *halotolerans* RFP74 was optimized using response surface methodology.

**Results:**

*Bacillus halotolerans* RFP74 inhibited the growth of various phytopathogens including *Alternaria* and *Bipolaris* with an inhibition rate of more than 60%. In addition, it also demonstrated an essential production of amylase. Based on previous studies of amylase production in *Bacillus*, three parameters were considered significant: initial pH of the medium, incubation time, and temperature. Using the central composite design with Design Expert software, the optimized amylase production for *B.* *halotolerans* RFP74 is at a temperature of 37 °C, incubation time 51 h and pH 6.

**Conclusion:**

The biological control agent *B.* *halotolerans* RFP74 inhibited the growth of *Alternaria* and *Bipolaris*, demonstrating its broad spectrum of activity. Knowledge of the optimal condition required for the production of hydrolytic enzymes such as amylase provides information on the most effective application of this biological control agent.

## Background

Plant pests have long been a source of concern in global agriculture [27]. According to FAO [6], they cause a 40% annual loss in global agricultural production. Chemical pesticides have been used on crops to control these pests, but their use has become excessive over time, causing environmental and health issues [18]. Among these issues are the emergence of increasingly resistant germs, an imbalance in the composition of soil microflora, and the presence of chemical residues in food products [24].

Microorganisms with antagonistic capacity against these pests are seen as a solution to this problem [28]. *Bacillus* spp. are among the most common and widely used microorganisms in this field [17]. This is due to their ability to produce a variety of antimicrobial peptides, including fengycin, surfactin, and iturin. They also produce hydrolytic enzymes that affect the permeability of fungi cell walls, causing them to lyse [23].

*Bacillus* spp. are common in the environment, particularly in soil. They have the unique ability to produce spores aerobically when conditions become unfavourable [15]. This enables them to withstand harsh environments. *B. halotolerans* belongs to the *B. subtilis* group as well as the *B. mojanvensis* subgroup [3]. As a result, *B. halotolerans* has the same phenotypic characteristics as the *B. subtilis* group. They are aerobic, Gram-positive, rod-shaped, mobile bacteria with ellipsoidal spores located centrally or paracentrally [11]. *B. halotolerans*, like the other species in the *B. subtilis* group, is frequently isolated from soil, water, and air. These microorganisms are regarded as non-pathogenic because they do not cause disease [14].

Biopesticides, such as bacteria with antifungal properties, are currently in high demand due to the environmental hazards of chemical pesticides [8]. To inhibit phytopathogens, they use a variety of mechanisms, including the production of hydrolytic enzymes such as amylase [22]. The biosynthesis of these enzymes is partially dependent on the growth of the producing strain [12]. The latter is usually determined by variables such as temperature, pH, and incubation time. Many *Bacillus* species have similar growth patterns, though strains may differ in general properties and optimal fermentation conditions [5]. Finding the optimal conditions for amylase production by RFP74 may take some time, depending on the number of tests that must be performed for each factor [19]. The most important factors influencing amylase production in *Bacillus* species were determined in this study, and then a design of experiments was established using Design Expert® software. Each factor and its interactions were optimised using the response surface methodology and the Central Composite Design. The goal of this study is to determine the optimal values of the most important factors for amylase production in *B. halotolerans* species. Based on our research, this the first time that the optimization of the production of amylase in *B.* *halotolerans* species is reported. 

## Methods

### Origins of bacterial and fungal isolates

*B. halotolerans* strain RFP74 was isolated from tomato rhizosphere in Oran, west of Algeria in 2019 [16]. It has been evaluated as a biocontrol agent against fungal phytopathogens: *Fusarium* *oxysporum* f.sp. *lycopersici* and *Ascochyta* spp.

The fungal phytopathogens used in this study are *Alternaria tomatophila, A. alternata, A. solani* and *Bipolaris* spp. These fungi belong to the collection of Es Senia Laboratory of Applied Microbiology.

The microorganisms were subcultured into fresh growth media, which included nutritive agar for bacteria and PDA (Potato Dextrose Agar) for fungi strains. 

### Assessment of the antifungal activity of *B. halotolerans* RFP74

The antagonistic activity of RFP74 against plant pathogenic fungi was assessed using the dual culture method described by Oldenburg et al. [13]. A Petri dish (90 mm in diameter) containing 15 ml of potato dextrose agar (PDA) media was used. A 5-mm disk of the fungal pathogen taken from 7 days old cultures was deposited to the centre of the plate. On the peripherals of the Petri dish, 25 mm away from the fungal disk, the antagonistic bacterium was then spotted. For each fungal pathogen, a plate containing only the disk of mycelia was used as a control. The plates were incubated at 27 °C for 7 days. Each test was conducted in three replicates. In order to determine the inhibition rates, the following formula was applied:

$$\mathrm{Inhibition}\;\mathrm{rates}\;(\%)=\frac{\mathrm{diameter}\;\mathrm{of}\;\mathrm{the}\;\mathrm{colony}\;\mathrm{in}\;\mathrm{control}\;-\;\mathrm{diameter}\;\mathrm{of}\;\mathrm{the}\;\mathrm{colony}\;\mathrm{in}\;\mathrm{treatment}}{\mathrm{diameter}\;\mathrm{of}\;\mathrm{the}\;\mathrm{colony}\;\mathrm{in}\;\mathrm{control}}\;\times\;100$$

### Detection of amylase production

The production of amylase was evaluated on starch medium (5.0 Peptone, 5.0 NaCl, 1.5 yeast extract, 1.5 meat extract, 2.0 starch, 15 agar all values in g/l). A single colony from a young culture of RFP74 was spotted to the centre of a Petri dish containing starch medium. Plates were incubated at 30 °C for 24 h. After the incubation, an iodine solution was poured into the plate until the colony was submerged. It was then left for 1 min. The excess iodine was poured. The result was considered positive if a clear halo was observed around the colony. The area away from the colony appears navy blue due to the reaction of iodine with starch. The test was conducted in three replicates.

### Enzyme activity assay

To determine the enzymatic activity, the method outlined by Tanyildizi et al. [29] was followed. Fifty milliliters of the prepared starch media were added into an Erlenmeyer of 100 ml. The pH was adjusted according to the required conditions.

Then, the media were inoculated with the bacterial strain and incubated according to the required parameters listed in (Table 1), in a static condition. After this incubation phase, the enzyme activity was evaluated. This involved centrifuging the cultures at 7000 rpm (revolutions per minute) for 15 min at 4 °C. Then, 0.5 ml of supernatant was taken and added into 4.5 ml of a substrate solution (Starch (3%) 2.3 ml; CaCl2 (0.1N) 1 ml; phosphate buffer (pH 6.2) 250 ml; NaCl 0.025N; distilled water 200 ml). For the control, 0.5 ml of uninoculated starch medium was added into 4.5 ml of substrate solution. Both solutions were incubated at 37 °C for 30 min. After this incubation phase, 0.9 ml of HCl (1N) was added to stop the reaction, then 0.1 ml of iodine solution and 4 ml of distilled water were added respectively. Finally, the optical density was measured at 620 nm.

**Table 1:** List of factors and their levels

Factors	− 1	0	+ 1
A: Temperature (°C)	25	40	55
B: Incubation time (hours)	24	48	72
C: Initial pH	6	8	10

A unit of enzymatic activity is defined at OD = 0.00284 of the reduction of the blue colour of the starch solution at 37 °C.

### Optimization of amylase activity

To optimize the production of amylase in RFP74, the most important factors as well as their maximum and minimum values have to be determined. For this purpose, several similar studies were consulted [1, 7, 21, 25, 30]. Among the factors that were observed, the three that were considered significant are temperature, incubation time and ph. The Response Surface Methodology is by definition a statistical tool that follows mathematical combinations and statistical techniques in order to study the effects of variables in enzyme production. The Central Composite Design (CCD) was used for the optimization of amylase production. The experimental design was produced using the Design Expert® software (Version 13.0.0). 

## Results

### In vitro effect of *B. halotolerans* RFP74 on the growth of plant pathogens *Alternaria* spp. and *Bipolaris* spp.

**Fig. 1:**
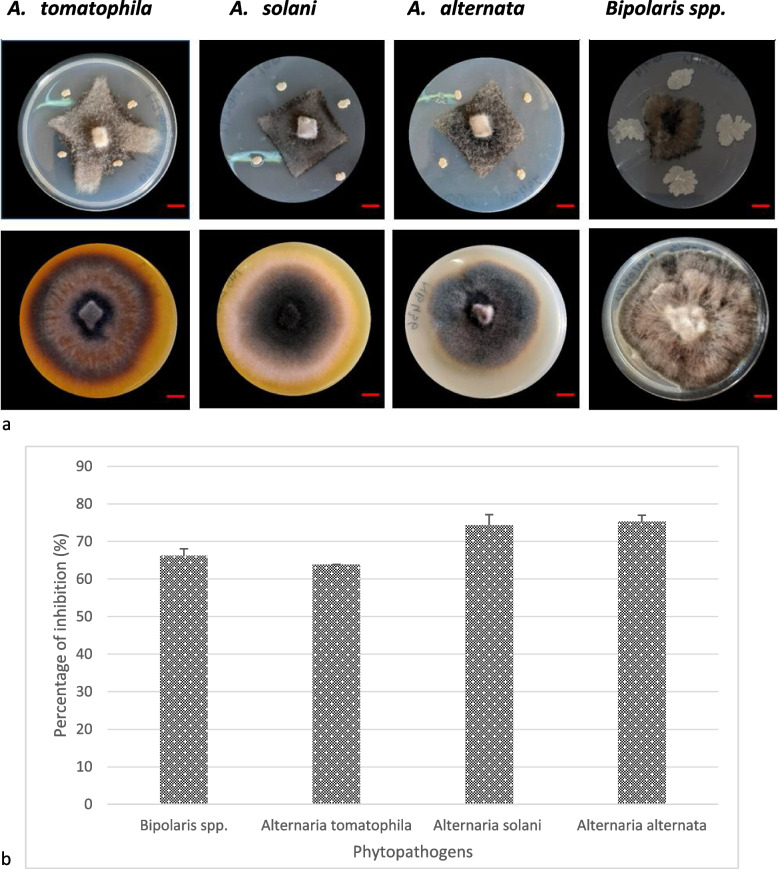
Antifungal activity of *Bacillus* *halotolerans* RFP74. **a** Dual culture assay of *B.* *halotolerans* RFP74 against multiple plant pathogens: *A. tomatophila*, *A. solani*, *A. alternata*, and *Bipolaris* spp. The upper figures depict the fungi with bacteria spotted in four peripheries, while the lower figures depict the control with only the fungus and no bacteria. Scale bar is 10 mm. **b** Inhibition rates of the plant pathogens by *B.* *halotolerans* RFP74

In a previous study [16], the efficacy of RFP74, not only to inhibit the growth of plant pathogens such as *Fusarium* *oxysporum* and *Ascochyta*, but also to promote plant development, was demonstrated. In this study, using a dual culture method, strain RFP74 showed antagonistic activity against some of the most problematic plant pathogenic fungi for crops in North Africa: *Bipolaris* spp. and three different species of *Alternaria* (*A.* *tomatophila, A. alternata* and *A.* *solani*). RFP74 affected fungi growth and appearance (Fig. 1a). In addition, when calculated, inhibition rates for all fungal pathogens were greater than 60% (Fig. 1b).

### Amylase production

**Fig. 2:**
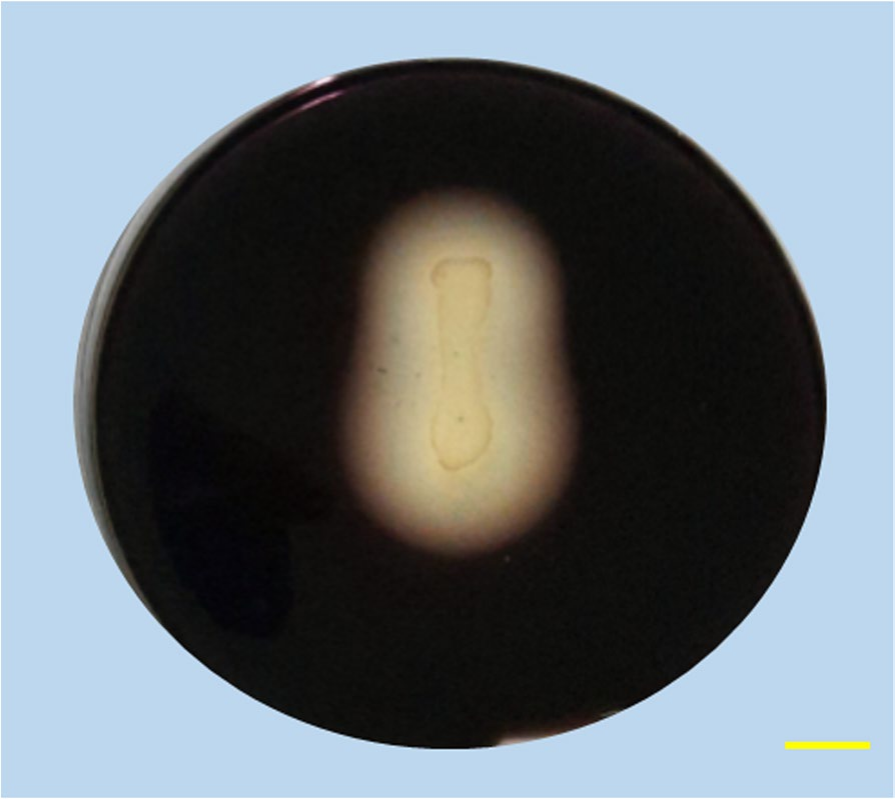
Amylase production by *B. halotolerans* RFP74. The clear zone around the colony indicates the hydrolysis of the starch. Scale bar (in yellow) is 10 mm

The ability of the RFP74 bacterial isolate to produce amylase was obtained by the starch hydrolysis plate assay method. RFP74 was able to hydrolyse the starch by showing a zone of clearance around the colonies on the starch medium after being flooded with iodine solution (Fig. 2).

### Optimization of the amylase production by *B. halotolerans* RFP74

**Fig. 3:**
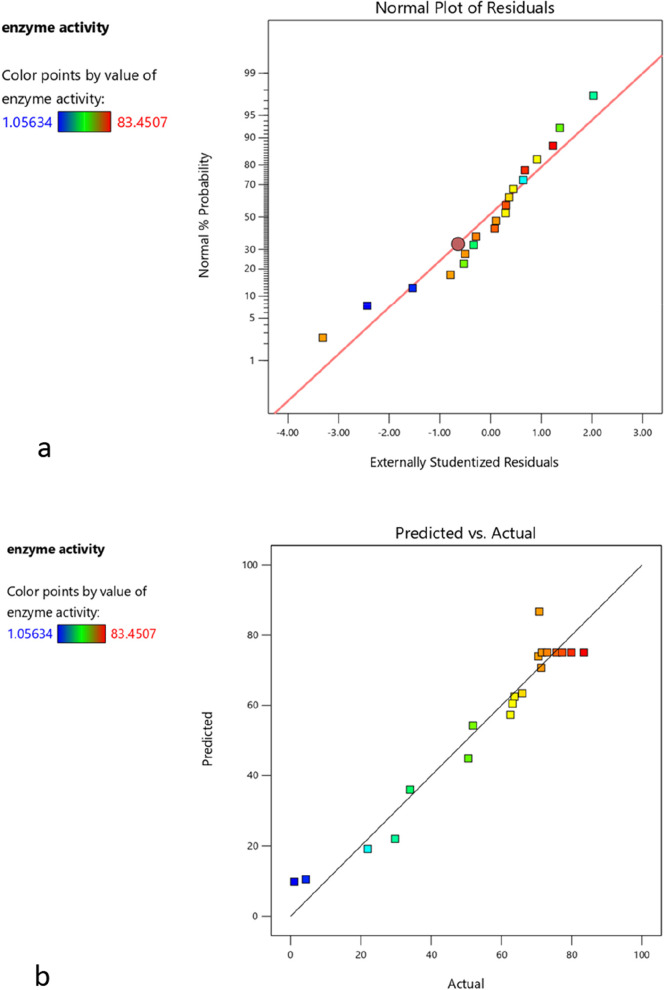
**a** Normal probability plot of Residuals. **b** Predicted and actual response slope of amylase production by *B.* *halotolerans* RFP74

To enhance the enzyme production by the strain RFP74, a response surface methodology model using the central composite design technique was designed to determine the levels of the most important parameters and their interaction effect. Initial pH, temperature, and incubation time were selected based on the results of previous studies that reported the crucial role of these parameters in the production of amylase in *Bacillus* species. For each of the three factors, two levels and a midpoint were determined (Table 1). According to the CCD methodology, 20 experimental runs were conducted and the amylase activities were considered as the response. Each test was performed in triplicates and the mean of these repetitions was taken as the response (Table 2). Based on the fit summary, the suggested model for the study was the quadratic model. The coefficient of determination (*R*^2^) was 0.9446, which means 94.46% of the data were well matched. Moreover, the adequate precision value, which measures the signal-to-noise ratio, was found to be 16.65 (Table 3). This ratio indicated an adequate signal that made the model suitable to navigate the design space (Fig. 3).

**Table 2:** Amylase activity as a response for each experiment

Run	Space type	Factor 1 A:temp (°C)	Factor 2 B:time (h)	Factor 3 C:pH	Amylase activity (UI)
1	Axial	55	48	8	34.0141
2	Center	40	48	8	71.5141
3	Factorial	55	24	10	21.9718
4	Axial	40	24	8	63.1338
5	Axial	25	48	8	62.5352
6	Axial	40	48	10	65.9507
7	Center	40	48	8	73.0634
8	Factorial	55	24	6	51.9366
9	Factorial	25	24	6	50.5634
10	Center	40	48	8	75.7042
11	Factorial	55	72	6	29.7183
12	Factorial	55	72	10	4.29577
13	Factorial	25	24	10	1.05634
14	Center	40	48	8	77.2887
15	Center	40	48	8	79.9296
16	Factorial	25	72	6	70.5282
17	Axial	40	72	8	71.338
18	Center	40	48	8	83.4507
19	Factorial	25	72	10	63.7676
20	Axial	40	48	6	70.8099

**Table 3:** Fit summary

Source	Sequential *p* value	Lack of fit *p* value	Adjusted *R*^2^	Predicted *R*^2^	
Linear	0.2192	0.0003	0.0921	− 0.4492	
2FI	0.2929	0.0003	0.1524	− 3.0241	
Quadratic	< 0.0001	0.0595	0.9122	0.7670	Suggested
Cubic	0.0321	0.3760	0.9686	− 0.9424	Aliased

The ANOVA analysis for the response surface quadratic model were summarized in Table 4. The statistical significance of the model equation and the model terms was determined by the *F* value which was found to be 29.22, this implies that the model is significant and there is only a 0.01% chance that an *F* value this large could occur due to noise. All seven terms included in this model were significant. These include linear, quadratic and interactions terms: temperature (*A*), incubation time (*B*), pH (*C*), the quadratic effects of temperature (*A*^2^), the quadratic effects of incubation time (*B*^2^), the interaction effects between temperature and time (AB), and the interaction effects between time and pH (BC).

**Table 4:** ANOVA analysis

Source	Sum of squares	df	Mean square	*F* value	*p* value	
Model	11,040.21	7	1577.17	29.22	< 0.0001	Significant
A-temperature	1134.53	1	1134.53	21.02	0.0006	
B-time	259.96	1	259.96	4.82	0.0486	
C-pH	1357.55	1	1357.55	25.15	0.0003	
AB	1877.94	1	1877.94	34.79	< 0.0001	
BC	279.53	1	279.53	5.18	0.0420	
A^2^	2594.22	1	2594.22	48.06	< 0.0001	
B^2^	289.50	1	289.50	5.36	0.0391	
Residual	647.76	12	53.98			
Lack of fit	550.40	7	78.63	4.04	0.0718	Not significant
Pure error	97.37	5	19.47			
Cor total	11,687.98	19				

The second order polynomial equation used for ANOVA:

$$\text{Amylase activity} \, \text{=} \, \text{75.12} \, - \, \text{10.65A} \, \text{+} \, \text{5.10B} \, - \, \text{11.65C} \, - \, \text{15.32AB} \, \text{+} \, \text{5.91BC} \, - \, \text{28.47}{\text{A}}^{2 } \, - \, \text{9.51}{\text{B}}^{2}$$

**Fig. 4:**
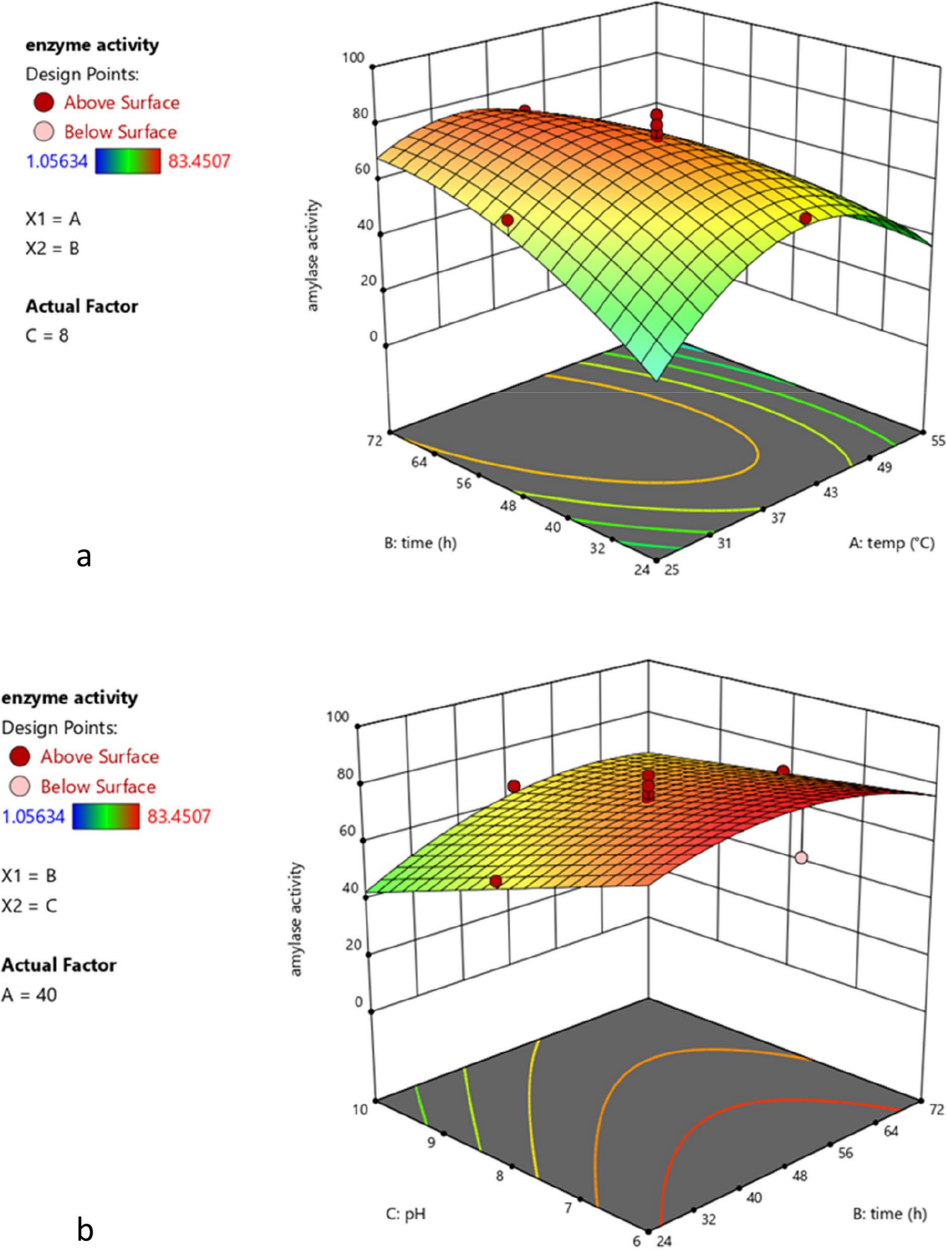
3D surface plot of amylase activity by *B.* *halotolerans* RFP74 (**a**) as a function of the interaction between incubation time and temperature with a pH set at 8 (**b**) as a function of the interaction between pH and incubation time with a temperature set at 40 °C

To obtain 3D responses in the CCD design for the evaluation of the interaction between variables, two criteria were used. First, the interaction between time and temperature was evaluated with the pH value set at 8. Figure 4a shows that when the temperature and incubation time increase, the amylase activity also increases. However, when the optimal values of these parameters were exceeded, the amylase activity decreased. Optimum temperature values were 35–45 °C and 48–51 h for incubation time.

Second, the effect of interaction between time and pH values on amylase production by RFP74 at a fixed temperature of 40 °C is shown in Fig. 4b. Amylase activity increased over time until optimal values were reached, and then began to decrease. This is likely due to nutrient reduction, waste accumulation, cell death and catabolic suppression [2, 21]. The maximum production of amylase was found at a pH between 6 and 8. The increase in pH values caused a decrease in amylase activity. The high pH value certainly influenced the growth of the bacterium and its metabolic activity since the ideal pH range for *B.* *halotolerans* growth is between 6 and 8 [16]. The results of this CCD optimization showed that to achieve maximum amylase production in RFP74 the temperature must be 37 °C, the pH value at 6 and the incubation time for 51 h.

## Discussion

The ability to inhibit multiple plant pathogens can play a major role in the potential use of RFP74 as biopesticides. As indicated by Spadaro and Gullino [26], the specificity of the action of a biological agent is very important from an environmental point of view, but for farmers who need to control several pathogens at the same time, this could be disadvantageous. In this case, having a biological agent like RFP74, capable of controlling several plant pathogens at the same time, can be a solution. Sagredo-Beltrán et al. [20] also reported similar results: *B.* *halotolerans* MS50-18A inhibited plant pathogens such as *Phytophthora capsici*,* Fusarium* *solani*,* Rhizoctonia solani*, and *Fusarium* *oxysporum*. The inhibition rate against these phytopathogens has been estimated at over 60%. Another recent study, Li et al. [10], reported 21–34 mm inhibition diameters against eight different plant pathogens, including *Botrytis* *cinerea*,* Fusarium graminearum*,* Curvularia lunata*, and *Phytophtora nicotianae*, using the dual culture method.

The production of hydrolytic enzymes is one of the most useful mechanisms used by biological control agents to inhibit the growth of plant pathogens. Most studies on the role of extracellular enzymes in biocontrol focus on enzymes such as protease, chitinase, and cellulase because of their ability to hydrolyse proteins, chitin, and cellulose, respectively. Since the fungal cell wall is mainly composed of chitin and cellulose, the degradation caused by these enzymes will lead to cell wall lysis. Unlike cellulase and chitinase, amylase degrades starch and is currently used more in industrial production. However, in recent years, the role of amylase enzymes in inhibiting phytopathogenic fungi has been highlighted. Huang et al. [9] demonstrated that the extracellular gene amylase is involved in the colonization capacity of *B. cereus* 0–9. By colonizing its environment, the biological control agent limits the availability of space and nutritional resources to plant pathogens, which causes their inhibition and thus prevents plant diseases. In addition, amylases can play a role in promoting plant growth by degrading starch in the soil [4]. Amylases break down complex polysaccharides like starch into simple sugars or glucose that are easily absorbed by plants and promote growth. Therefore, by producing amylases, RFP74 not only inhibits phytopathogens but also promote the growth of plants.

The present study aimed to investigate and optimize the production of amylase by *B.* *halotolerans*, using statistical method. Our results showed that RFP74 was able to produce a significant amount of extracellular amylase when grown in a medium containing starch as the sole carbon source. The maximum enzyme activity was observed in the stationary phase of growth (51 h), indicating that amylase production is closely related to cell growth.

Our study demonstrated that RFP74 is a promising candidate for amylase production, which enhance their potential use as a biocontrol agent against fungal phytopathogens. The bacterium exhibited optimal amylase production at 37 °C, pH value at 6 and 51 h of incubation time.

## Conclusion

This study focused on optimizing amylase production by an extremely important bacterial strain since, in a previous study, they have shown great potential for use as a biological control agent against some of the plant pathogens frequently found in North Africa. Using the Response Surface Methodology model, we demonstrated that to achieve maximum amylase production, these parameters should be defined: temperature at 37 °C, incubation time of 51 h, and pH at 6. Given the importance of amylase as one of the mechanisms used by biocontrol agents to prevent plant diseases and promote plant growth, this study will serve as a reference on how to maximize the production of this enzyme, particularly for those working on the *B.* *halotolerans* species.

## Data Availability

Not applicable
